# The Longitudinal Association Between Preadolescent Facial Emotion Identification and Family Factors, and Psychotic Experiences in Adolescence (The TRAILS Study)

**DOI:** 10.1007/s10578-019-00922-4

**Published:** 2019-09-04

**Authors:** Laura A. Steenhuis, Gerdina H.M. Pijnenborg, Elisabeth C.D. van der Stouwe, Catharina A. Hartman, André Aleman, Agna A. Bartels-Velthuis, Maaike H. Nauta

**Affiliations:** 1 Department of Clinical Psychology and Experimental Psychopathology, Faculty of Behavioural and Social Sciences, University of Groningen, Grote Kruisstraat 2/1, 9712 TS Groningen, The Netherlands; 2 Department of Psychotic Disorders, GGZ Drenthe, Assen, The Netherlands; 3 Department of Neuroscience, University Medical Center Groningen, University of Groningen, Groningen, The Netherlands; 4 Interdisciplinary Center Psychopathology and Emotion Regulation, Department of Psychiatry, University Medical Center Groningen, University of Groningen, Groningen, The Netherlands; 5 Rob Giel Research Center, University Center for Psychiatry, University Medical Center Groningen, University of Groningen, Groningen, The Netherlands

**Keywords:** Adolescence, Psychosis, Parenting, Family functioning, Social cognition

## Abstract

The current study examines whether facial emotion identification and family factors at preadolescence (age 11) predict psychotic experiences 5 years later during adolescence (age 16) and whether family factors may mediate the association between facial emotion identification and psychotic experiences. Data was obtained from the epidemiological cohort TRAILS (N = 2059). At preadolescence, a facial emotion identification test and three questionnaires to assess family functioning, perceived parenting styles and parenting stress, were administered. At adolescence, a questionnaire on psychotic experiences was administered. Facial emotion identification at preadolescence was not associated with psychotic experiences at adolescence, and the mediational role of family functioning was not further explored. However, increased overprotective parenting at preadolescence was associated with a higher frequency of psychotic experiences and delusions at adolescence. Future research may examine the mechanism behind the role of overprotective parenting on psychotic experiences during adolescence.

## Introduction

Psychotic disorders have often been associated with social cognitive impairments [1]. One of the domains of social cognition is facial emotion identification [2], which refers to the ability to accurately identify emotional expressions from another person’s face. The ‘basic’ set of emotions (anger, disgust, fear, sadness, surprise and happiness) as proposed by Ekman and colleagues are each characterized by a distinct facial expression, physiology and evolutionary purpose [3]. The ability to accurately recognize these emotions is crucial in facilitating emotional connections and communicating effectively with others. In psychotic disorders, recognition of positive expressions (happiness) is preserved and recognition of negative expressions (anger, fear, sadness and disgust) is impaired [4–7], although some studies report impairments for both positive and negative emotions [8].

Recent studies demonstrated that impairments in the identification of facial affect are not only found in chronic psychosis [9], but also in first episode psychosis [10], the ultra-high risk phase of psychosis [11, 12], and in siblings [13]. The evidence suggests that early impairment may show up for specific emotions, rather than as a general deficit [10]. Deficits in facial emotion identification have been hypothesized to play a role in the development of psychotic experiences. To specify, facial emotion identification deficits could give rise to paranoia (an inability to understand others could feed negative interpretations [5, 14]), delusions (an inability to correct faulty interpretations can cause and support delusional ideation [15]), and potentially hallucinations (continuous erroneous interpretation of social situations and others can lead to social stress, hyper vigilance, and hallucinatory experiences [16] (see also a review by [17]). Overall, an impairment in facial emotion identification may be a trait vulnerability for psychosis, rather than a consequence of the disorder. It is important to investigate at which point facial emotion identification impairments can be ‘detected’ as to examine when early interventions may be possible and effective. Given that psychotic experiences are prevalent in samples of youth [18–20], and may signify a precursor to psychotic disorders [21], it is fruitful to examine whether reduced facial emotion identification in preadolescence is associated with psychotic experiences during adolescence.

Social cognitive impairments have been found to contribute to diminished social functioning in psychotic disorders [22]. If deficits in facial emotion identification are present from childhood, this may already lead to problems in the development of socially competent behaviors and interactions. Given the importance of the family environment for children and adolescents’ functioning [23], it is possible that children with poor facial emotion identification skills have more difficulty functioning in the family environment as well. For example, children with poor facial emotion identification skills may perceive parenting as more negative, either due to inaccurate identification of emotions of their parents, or due to an accurate perception of more rejective or overprotective parenting as a reaction to their lower social cognitive abilities. Therefore, if facial emotion identification abilities predict psychotic experiences throughout adolescence, it may be especially interesting to explore the possibility whether this association might be mediated, at least partially, by family functioning.

The family context has gained much attention in psychosis studies, mostly in more acute and chronic phases of illness [24–30]. There is a strong indication that family factors such as expressed emotion [24, 27], the family rearing environment [25, 28] and family communication [26, 30] are important predictors of the prognosis of psychosis once an individual has transitioned to a first psychotic episode. Several prospective studies have found that patients with family members who are high in expressed emotion (over-involvement, high criticism, and negative affective style) are at an increased risk of relapse in schizophrenia over a period of nine to 12 months [24, 31]. Also in children with elevated mental health problems, parental styles (such as communication deviance, expressed emotion or affective style), significantly predicted schizophrenia spectrum disorders in adulthood in a 15 year prospective longitudinal study [26], although it is important to note that this sample was limited in its size (n = 50). In the ultra-high risk phase of psychosis (before the first psychotic episode), family functioning (a positive warm environment) has been shown to be protective [32, 33], both for reducing negative and disorganized symptoms, and improvement in functioning over a period of 3 [32] and 6 [33] months. Whether the family environment and parenting styles are predictive of the development and course of psychotic experiences (rather than a reaction towards clinical symptoms) during adolescence, remains understudied so far.

The aim of the current study is to examine whether a) facial emotion identification and b) family factors at preadolescence (age 11) predict psychotic experiences 5 years later during adolescence (age 16). We expect that both lower facial emotion identification abilities and more negative family functioning in preadolescence will predict a higher frequency of psychotic experiences at adolescence. If confirmed that facial emotion identification abilities are associated with psychotic experiences in adolescence, we will further explore whether functioning in the family environment (at least partially) mediates the relationship between facial emotion identification and psychotic experiences. Given that childhood mental health is associated with parenting behaviors at preadolescence [34] and is likely to predict adult mental health, the current study will control for pre-adolescent mental health problems (internalizing and externalizing behaviors).

## Methods

### Sample

Data used in the current study were collected as part of the longitudinal ‘TRacking Adolescents Individual Lives Survey’ (TRAILS), a prospective cohort study which aims to elucidate the etiology of mental health problems during adolescence [35, 36]. The National Dutch Medical Ethical Committee approved this study and the research has been performed in accordance with the ethical standards laid down in the 1964 Declaration of Helsinki and its later amendments. Written informed consent was obtained from all adolescents and their parents in this study. As done in previous studies in this cohort [37], we merged data from two TRAILS samples, a large population-based birth cohort (n = 2230) and a smaller parallel clinic-referred cohort (n = 543), in order to acquire a large sample with a wide variation in mental health. Data of the first and third data collection waves with mean ages of around 11 (T1) and 16 (T3) years were used for the current study. Participants were included if they at least completed the assessment on psychotic experiences at T3 (n = 2059). Due to missing data, N varies between 1956 and 2059 in the total sample.

Full details on the sampling procedure, descriptive statistics, response rates and selective attrition have all been provided in previous studies [36, 38]. In summary, to obtain the population cohort, TRAILS approached 135 primary schools in five municipalities in the north of the Netherlands, of which 90.4% agreed to participate. After contacting eligible preadolescents and their parents, 2230 participants (76% of those that were contacted) were enrolled in the study at T1 (mean age = 11.1 years, SD = .0.56; 49.2% boys). Five years later, 81% of them participated at T3 (N = 1816; mean age, 16.3 years, SD = .0.7; 48% boys). The two data waves included in this study ran from March 2001 to July 2002 (T1), and from September 2005 to August 2007 (T3). The clinic-referred cohort contained preadolescents who had been referred to the Groningen University Child and Adolescent Psychiatric Outpatient Clinic at any point in their life. At T1, 543 participants (43% of those that were contacted) participated in the study (mean age = 11.1 years, SD = 0.50; 65.9% boys). In total 416 (76.6%) of them completed measurements at T3. The data waves in the clinic-referred cohort started 2 years after the population cohort: from September 2004 to December 2005 (T1), and from and September 2009 to February 2011 (T3). The same design and instruments were used for both cohorts.

### Measures

To assess *facial emotion identification* at T1, we used the Identification of Facial Expressions Task, which is part of the Amsterdam Neuropsychological Tasks program (ANT [39]). This task is a reliable and valid instrument with acceptable test–retest reliability, and construct, criterion, and discriminant validity [39–41]. Trained undergraduate psychologists assessed each participant individually. The task consists of six parts of 40 trials each, divided over 20 target and 20 non-target trails. Each part focusses on a specific emotion (happy, sad, angry, fear, surprise and disgust) and lasts 5 min in total. Participants were instructed to press the yes-button for a target emotion and the no-button if a different emotion was displayed. For our study, we selected all emotions except surprise, as we focused explicitly on positive and negative emotions and surprise is considered as neither positive nor negative [42]. Each emotion was examined separately, as early impairment may show up for specific emotions, rather than a general deficit [10]. For each emotion the error proportion (EP) and reaction time (RT) was calculated. EPs were calculated as the mean proportion of misses and false alarms, using the subsequent equation: EP = ((misses/(misses + hits)) + (false alarms/(false alarms + correct rejections)))/2. RTs were calculated by the mean RT across hits and correct rejections. EPs and RTs that were more than four standard deviations above the mean [43] as well as participants performing at chance level of accuracy (50% or more errors) were considered missing [44]. In addition, outliers in one outcome parameter were also noted missing for the other, as EP and RT may influence each other. For each emotion, standardized Z-scores were created for both the RTs and EPs. It is important to examine both the EPs and RTs of emotions, as both aspects could reveal distinct and independent associations with the development of psychotic experiences [45]. Therefore, 10 variables of facial emotion identification were constructed: EP happy, EP sad, EP angry, EP fear, EP disgust, RT happy, RT sad, RT angry, RT fear and RT disgust.

To assess *family functioning* at T1, a modified version of the General Functioning Scale of the McMaster Family Assessment Device (FAD; [46]) was administered to the primary parent. The FAD has shown to have adequate test–retest reliability, good divergent and convergent validity, in addition to adequate sensitivity and specificity [47]. The scale includes six dimensions of family functioning, consisting of communication, problem solving, affective responsiveness, affective involvement, roles and behavior control. The scale comprises twelve items with a 4-point scale, ranging from 1 (totally disagree) to 4 (totally agree). A sum score was computed by adding up all items (a higher score indicates lower family functioning).

To assess *perceived parenting style* at T1, the EMBU-C [48] was administered, which is the child version of the EMBU (English translation: My Memories of Upbringing; [49]). The EMBU-C has good psychometric properties and convergent validity [48]. The questionnaire contains the following three scales: Rejection (12 items), Emotional Warmth (18 items), and Overprotection (12 items). Items are assessed using a 4-point scale, ranging from 0 (no, never) to 4 (yes, almost always). Responses of fathers and mothers were highly correlated for rejection (*r* =0 .68, *p* <0.001), emotional warmth (*r* =0 .79, *p* < 0.001) and overprotection (*r* =0 .81, *p* < 0.001), and therefore, in line with previous TRAILS papers [34, 50–52], scores were combined (averaged) for both parents. If information for only one parent was present, the score for the one parent was used.

To assess *parental stress* at T1, a short Dutch form of the Parental Stress Index (PSI [53]) was administered. The Dutch version has been found to have good psychometric properties and construct validity [54]. It is a 25-item questionnaire to assess the magnitude of stress in the parent–child relationship. Items are rated by the parent on a 6-point scale from 1 (disagree very much) to 6 (agree very much). The instrument contains two subscales, assessing the child’s characteristics (11 items) and the parents’ characteristics in the parenting context (14 items). A previous study [55] conducted a factor analysis of this measure in the current TRAILS sample, and discovered that one item did not load on either the child or the parent factor (item 24: “I feel confident about the future upbringing of my child”). Therefore, this item was excluded in the TRAILS cohort. For the purpose of this study, only the parent subscale was used to obtain a measure of perceived stress for the parent.

To assess *childhood mental health* at T1, the Youth Self-Report (YSR [56]) was administered. The YSR has a good test–retest reliability and discriminative validity [56]. In this 112-item questionnaire, descriptions of emotions and behaviors are rated on a three-point scale (not true (0), somewhat or sometimes true (1) and very often true (2)). These items assess two broad dimensions of behavior problems: internalizing (anxious/depressed, withdrawn/depressed and somatic complaints) and externalizing (aggressive behavior and rule-breaking behavior) problems. For the current study, a total score of all problem behaviors was computed based on 105 items (in line with [57]).

To assess *psychotic experiences* at T3, the Community Assessment of Psychic Experiences CAPE [58, 59] was used. The CAPE is a self-report questionnaire with good psychometric properties, discriminative validity [60] and test–retest reliability [58]. The positive experiences subscale has 20 items assessing the frequency and distress of positive experiences (e.g. delusions and hallucinations) separately. The frequency/distress of each item is assessed on a four-point scale [(1) never/no distress, (2) sometimes/a bit distressed, (3) often/quite distressed, and (4) nearly always/very distressed). For the current study, the frequency of positive experiences was used. Based on a factor analysis [61]] a previous study found five underlying dimensions of the CAPE that are differently associated with risk of future psychopathology. Their study [61] demonstrated that hallucinations, delusions and paranoia, but not grandiosity and paranormal beliefs, were mostly associated with distress and future psychopathology. For the current study these three risk sub-domains were separately identified by calculating a sum score of delusions (8 items) and paranoia (5 items), and a categorical score of hallucinations as either absent or present (0/1). Given the low endorsement rate of hallucinations in this sample, adolescents received a ‘present’ score on the hallucination variable if they endorsed at least one (or more) of the three hallucination items.

### Statistical Analyses

Analyses were carried out in SPSS 25. To examine whether the hypothesized predictors were related to the outcomes of our study, Pearson’s correlations were first computed between facial emotion identification variables (RTs, EPs), psychotic experiences (total frequency, hallucinations, delusions and paranoia), and family factors (family functioning, overprotective, warm and rejective parenting, and parental stress). With the relevant associations identified, a number of multiple linear and logistic regression models were run to examine our hypotheses in a step-wise approach. All assumptions of these analyses (e.g. homoscedasticity and normality of residuals) were checked beforehand. First, psychotic experiences (age 16) were predicted by facial emotion identification variables (age 11) (linear and logistic regression models). Second, psychotic experiences (age 16) were predicted by family factors at preadolescence (age 11) (linear and logistic regression models). Third, family factors were predicted by facial emotion identification (both at age 11) (linear regression models). Findings were corrected for multiple testing with the Bonferroni-Holmes correction, thus correcting the *p* value per step off, starting with the lowest *p* value [62]. All analyses were controlled for age, sex and pre-adolescent mental health problems. If our first hypothesis was met, we aimed to explore whether family functioning (age 11) mediates the relationship between the relevant facial emotion identification variable (age 11) and psychotic experiences (age 16). This was done with the computational process PROCESS [63], for which a ‘parallel multiple mediation model’ was computed, where X (the causal variable: facial emotion perception), was modeled to influence Y (the outcome variable: psychotic experiences) directly, as well as indirectly, through multiple mediator variables (the mediators: family functioning, overprotective, warm and rejective parenting, and parental stress).

## Results

### Descriptives

Characteristics of the sample and assessments outcomes are given in Table 1. In the identification of facial emotions task, positive emotions were easier to recognize than negative emotions, as denoted by lower reaction times (t(2641) = 76.32, p <0 .01) and lower proportion of errors (t(2641) = 57.29, p <0 .01). In Table 2, correlations between all variables are displayed.

**Table 1:** Characteristics of the sample and assessments outcomes

	N	Mean (SD)/frequency (%)	Range
Age	2059	16.17 (0.69)	14.42–18.36
Sex (% female)	2059	1018 (49.4)	
T1			
Mental health (YSR; total problems)	2018	0.36 (0.19)	0.00–1.18
Facial emotion recognition (IFE task)			
EP			
Happy	2030	3.31 (3.45)	0–17.50
Sad	2033	12.88 (9.41)	0–45
Angry	2020	8.34 (6.18)	0–35
Fear	2020	7.74 (6.84)	0–37.50
Disgust	2026	6.16 (5.53)	0–30
RT			
Happy	2030	880 (206)	458–1750
Sad	2033	1210 (286)	528–2449
Angry	2020	1116 (257)	581–2188
Fear	2020	1113 (280)	552–2323
Disgust	2026	1062 (250)	546–2091
Family functioning (FAD)	1956	1.79 (0.38)	1.00–4.00
Parental stress (PSI)	1959	1.93 (0.89)	1.00–5.60
Parenting behavior (EMBU-C)			
Warm parenting	2047	3.22 (0.49)	1.17–4.00
Rejective parenting	2046	1.51 (0.32)	1.00–3.44
Overprotective parenting	2046	1.86 (0.37)	1.00–3.44
T3			
Psychotic experiences (CAPE)			
Total frequency	2059	1.28 (0.23)	1.00–2.85
Hallucinations (N, %)	2051	299 (14.6)	
Delusions	2037	1.20 (1.73)	0.00–17.00
Paranoia	2039	2.61 (1.77)	0.00–10.00

T1 Age 11, T3 Age 16

*IFE* identification of facial expressions task, *EP* error percentage (raw), *RT* reaction time (raw), *FAD* family assessment device, *PSI* parental stress index, *EMBU*-*C* my memories of upbringing, *CAPE* community assessment of psychic experiences

**Table 2:** Correlations between psychotic experiences (frequency, hallucinations, delusions and paranoia), facial emotion identification (happy, sad, angry, fear and disgust) and family factors (family functioning, parental stress, warm, rejective and overprotective parenting)

Questionnaire/task	Variables	1.	2.	3.	4.	5.	6.	7.	8.	9.	10.	11.	12.	13.	14.	15.	16.	17.	18.	19.
IFE task	1. RT happy	–	–	–	–	–	–	–	–	–	–	–	–	–	–	–	–	–	–	–
	2. RT sad	0.68**	–	–	–	–	–	–	–	–	–	–	–	–	–	–	–	–	–	–
	3. RT angry	0.65**	0.72**	–	–	–	–	–	–	–	–	–	–	–	–	–	–	–	–	–
	4. RT fear	0.60**	0.64**	0.71**	–	–	–	–	–	–	–	–	–	–	–	–	–	–	–	–
	5. RT disgust	0.61**	0.67**	0.71**	0.73**	–	–	–	–	–	–	–	–	–	–	–	–	–	–	–
	6. EP happy	0.74**	− 0.04	− 0.03	− 0.04*	− 0.03	–	–	–	–	–	–	–	–	–	–	–	–	–	–
	7. EP sad	0.61**	0.10**	0.10**	0.09**	0.09**	0.29**	–	–	–	–	–	–	–	–	–	–	–	–	–
	8. EP angry	0.02	− 0.01	0.05*	0.05**	0.03	0.29**	0.43**	–	–	–	–	–	–	–	–	–	–	–	–
	9. EP fear	0.06**	0.03	0.04*	0.17**	0.07**	0.23**	0.36**	0.43**	–	–	–	–	–	–	–	–	–	–	–
	10. EP disgust	0.01	− 0.03	− 0.02	− 0.01	0.06**	0.28**	0.32**	0.38**	0.32**	–	–	–	–	–	–	–	–	–	–
FAD	11. Family functioning	0.02	0.02	0.01	0.01	0.03	0.44*	0.05*	0.03	0.01	0.04*	–	–	–	–	–	–	–	–	–
PSI	12. Parental stress	0.04*	0.04*	0.03	0.03	0.06**	0.01	0.07**	0.06**	0.07**	0.08**	0.43**	–	–	–	–	–	–	–	–
EMBU-C	13. Warm parenting	− 0.08**	− 0.06**	− 0.06**	− 0.05*	− 0.07**	− 0.03	− 0.09**	− 0.08**	− 0.05**	− 0.08**	− 0.15**	− 0.12**	–	–	–	–	–	–	–
	14. Rejective parenting	0.04*	0.04	0.04	0.02	0.06**	0.00	0.05*	0.02	0.02	0.03	0.12**	0.22**	− 0.32**	–	–	–	–	–	–
	15. Overprotective parenting	− 0.01	− 0.01	− 0.01	0.01	0.02	0.01	0.01	− 0.02	0.02	− 0.02	0.02	0.09**	0.19**	0.45**	–	–	–	–	–
CAPE	16. Total frequency	0.04	0.02	0.01	0.01	0.03	− 0.01	− 0.05*	− 0.05*	− 0.01	− 0.04	0.01	0.06^**^	0.03	0.07**	0.14**	–	–	–	–
	17. Hallucinations	0.06**	0.04	0.05*	0.03	0.05*	− 0.03	0.03	− 0.01	− 0.01	0.02	0.01	0.04*	− 0.02	0.04	0.07**	0.59**	–	–	–
	18. Delusions	0.04	0.03	0.01	0.01	0.03	0.03	− 0.01	− 0.01	0.00	0.00	0.02	0.05*	− 0.01	0.05*	0.11**	0.80**	0.48**	–	–
	19. Paranoia	0.04	0.01	0.00	− 0.00	0.01	− 0.04	0.06*	− 0.05*	− 0.01	− 0.04	0.02	0.05*	0.03	0.09**	0.13**	0.75**	0.28**	0.43**	–

N = 1956–2059

*IFE* identification of facial expressions task, *RT* reaction time (standardized), *EP* error proportion (standardized), *FAD* family assessment device, *PSI* parental stress index, *EMBU*-*C* my memories of upbringing, *CAPE* community assessment of psychic experiences

*p < .05, **p < .01

### Associations Between Facial Emotion Identification Abilities at Preadolescence (Age 11) and Psychotic Experiences at Adolescence (Age 16)

Facial emotion identification abilities at age 11 were not significantly associated with delusions at age 16, and thus not further examined in the regression models (see Table 2). Table 3 demonstrates both linear and logistic regression models, in which frequency of psychotic experiences, hallucinations and paranoia is predicted by facial emotion identification (EPs and RTs), after adjustment for confounders. The results demonstrate that facial emotion identification abilities at age 11 were not significantly associated with psychotic experiences at age 16. In the absence of an association, mediation by family factors was not explored.

**Table 3:** Results of linear and logistic regression analyses of psychotic experiences (frequency, hallucinations, delusions and paranoia) at age 16 on facial emotion identification reaction times at age 11

	Frequency of psychotic experiences					Hallucinations		Paranoia				
	B (95% CI)	SE B	β	t	p	OR (95% CI)	p	B (95% CI)	SE B	β	t	p
EP happy	0.00 (− 0.01 to 0.01)	0.01	0.01	0.48	0.63	0.92 (0.79 to 1.06)	0.25	− 0.01 (− 0.01 to 0.08)	0.04	− 0.00	− 0.15	0.88
EP sad	− 0.01 (− 0.02 to 0.00)	0.01	− 0.04	− 1.46	0.15	1.11 (0.96 to 1.30)	0.17	− 0.05 (0.14 to 0.04)	0.05	− 0.03	− 1.01	0.31
EP angry	− 0.01 (− 0.02 to 0.01)	0.01	− 0.03	− 0.99	0.33	0.93 (0.79 to 1.10)	0.39	− 0.04 (− 0.13 to 0.06)	0.05	− 0.02	− 0.74	0.46
EP fear	0.00 (− 0.01 to 0.02)	0.01	0.02	0.59	0.55	0.99 (0.84 to 1.16)	0.89	0.05 (− 0.05 to 0.14)	0.05	0.02	0.96	0.34
EP disgust	− 0.00 (− 0.01 to 0.01)	0.01	− 0.00	− 0.16	0.87	1.04 (0.90 to 1.21)	0.60	− 0.05 (− 0.13 to 0.04)	0.05	− 0.02	− 1.01	0.31
RT happy	0.01 (− 0.01 to 0.02)	0.01	0.03	0.91	0.36	1.29 (1.07 to 1.56)	0.01	0.14 (0.02 to 0.25)	0.06	0.07	2.31	0.02
RT sad	− 0.00 (− 0.02 to 0.02)	0.01	− 0.00	− 0.08	0.94	1.04 (0.84 to 1.29)	0.72	0.01 (− 0.13 to 0.14)	0.07	0.00	0.09	0.93
RT angry	− 0.00 (− 0.02 to 0.02)	0.01	− 0.01	− 0.23	0.82	0.99 (0.79 to 1.24)	0.94	− 0.06 (− 0.20 to 0.07)	0.07	− 0.03	− 0.92	0.36
RT fear	− 0.02 (− 0.03 to 0.00)	0.01	− 0.07	− 1.79	0.07	0.81 (0.65 to 1.02)	0.08	− 0.13 (− 0.26 to 0.00)	0.07	− 0.07	− 1.93	0.05
RT disgust	0.02 (0.00 to 0.04)	0.01	0.08	2.30	0.02	1.13 (0.91 to 1.40)	0.27	0.11 (− 0.02 to 0.24)	0.07	0.06	1.64	0.10

n = 2020–2059

All effects were adjusted for sex, age, and preadolescent mental health problems. p-values in bold indicate significance after Bonferroni–Holm correction

*OR* odds ratio, *RT* reaction time (standardized), *EP* error proportion (standardized), *CI* confidence interval

### Associations Between Family Factors at Preadolescence (Age 11) and Psychotic Experiences at Adolescence (Age 16)

Table 4 shows the results from four regression models (both linear and logistic) predicting psychotic experiences (frequency, hallucinations, delusions and paranoia) with family factors, after correcting for confounders. Findings demonstrate that overprotective parenting at age 11 was positively associated with both the frequency of psychotic experiences and delusions at age 16.

**Table 4:** Results of linear and logistic regression analyses of psychotic experiences (frequency, hallucinations, delusions and paranoia) at age 16 on family factors at age 11

	Frequency of psychotic experiences					Hallucinations		Delusions					Paranoia				
	B (95% CI)	SE B	β	t	p	OR (95% CI)	p	B (95% CI)	SE B	β	t	p	B (95% CI)	SE B	β	t	p
Family functioning (FAD)	− 0.01 (− 0.04 to 0.02)	0.02	− 0.02	− 0.78	0.44	1.18 (0.81 to 1.73)	0.40	− 0.04 (− 0.27 to 0.18)	0.11	− 0.01	− 0.38	0.70	− 0.02 (− 0.24 to 0.20)	0.11	− 0.00	− 0.18	0.86
Parental stress (PSI)	0.01 (0.00 to 0.03)	0.01	0.06	2.22	0.03	1.09 (0.93 to 1.28)	0.29	0.08 (− 0.02 to 0.18)	0.05	0.04	1.66	0.10	0.07 (− 0.03 to 0.17)	0.05	0.04	1.46	0.15
Warm parenting (EMBU-C)	0.01 (− 0.01 to 0.04)	0.01	0.03	1.19	0.23	0.73 (0.54 to 0.99)	0.05	− 0.09 (− 0.27 to 0.10)	0.09	− 0.03	− 0.94	0.35	0.19 (0.01 to 0.34)	0.09	0.05	2.03	0.05
Rejective parenting (EMBU-C)	− 0.03 (− 0.07 to 0.01)	0.02	− 0.04	− 1.42	0.16	0.77 (0.45 to 1.34)	0.35	− 0.25 (− 57 to 0.07)	0.17	− 0.05	− 1.51	0.13	0.02 (− 0.30 to 0.34)	0.16	0.00	0.14	0.89
Overprotective parenting (EMBU-C)	0.06 (0.02 to 0.09)	0.02	0.09	3.36	**0.00**	1.66 (1.08 to 2.55)	0.02	0.47 (0.21 to 0.72)	0.13	0.10	3.57	**0.00**	0.26 (0.01 to 0.51)	0.13	0.06	2.03	0.04

n = 2037–2059

All effects were adjusted for sex, age, and preadolescent mental health problems. p-values in bold indicate significance after Bonferroni–Holm correction

*OR* odds ratio, *CI* confidence interval, *FAD* family assessment device, *PSI* parental stress index, *EMBU*-*C* my memories of upbringing

### Associations Between Facial Emotion Identification Abilities and Family Factors at Preadolescence (Age 11)

Overprotective parenting was not significantly associated with family factors at age 11, and thus not further examined in the regression models (see Table 2). Table 5 shows the results from four linear regression models, predicting family factors (family functioning, parental stress, warm and rejective parenting) by facial emotion perception (EPs and RTs), after correcting for confounders. The results demonstrate that facial emotion perception abilities were not significantly associated with family factors at age 11.

**Table 5:** Results linear regression analyses of family factors on facial emotion identification reaction times at age 11

	Family functioning					Parental stress					Warm parenting					Rejective parenting				
	B (95% CI)	SE B	β	t	p	B (95% CI)	SE B	β	t	p	B (95% CI)	SE B	β	t	p	B (95% CI)	SE B	β	t	p
EP happy	0.00 (− 0.01 to 0.02)	0.01	0.01	0.48	0.63	− 0.05 (− 0.09 to 0.01)	0.02	− 0.05	− 2.4	0.02	0.01 (− 0.00 to 0.04)	0.01	0.03	1.57	0.12	− 0.01 (− 0.02 to 0.00)	0.01	− 0.03	− 1.85	0.07
EP sad	0.01 (− 0.00 to 0.03)	0.01	0.04	1.49	0.14	0.02 (− 0.02 to 0.06)	0.02	0.02	0.94	0.34	− 0.02 (− 0.05 to 0.00)	0.01	− 0.05	− 2.14	0.03	0.01 (− 0.00 to 0.02)	0.01	0.03	1.75	0.08
EP angry	0.01 (− 0.01 to 0.03)	0.01	0.02	0.72	0.47	0.02 (− 0.02 to 0.07)	0.02	0.03	1.07	0.29	− 0.02 (− .04 to .00)	0.01	− 0.04	− 1.73	0.08	0.01 (− 0.01 to 0.02)	0.01	0.02	0.80	0.42
EP fear	− 0.01 (− 0.03 to 0.01)	0.01	− 0.03	− 1.17	0.24	0.04 (0.00 to 0.09)	0.02	0.05	1.96	0.05	− 0.00 (− 0.03 to 0.02)	0.01	− 0.01	− 0.33	0.74	− 0.00 (− 0.02 to 0.01)	0.01	− 0.01	− 0.51	0.61
EP disgust	0.01 (− 0.01 to 0.02)	0.01	0.02	0.70	0.48	0.044 (0.00 to .09)	0.02	0.05	2.11	0.04	− 0.02 (− 0.05 to 0.00)	0.01	− 0.05	− 2.10	0.04	0.00 (− 0.01 to 0.02)	0.01	0.01	0.38	0.70
RT happy	0.01 (− 0.02 to 0.03)	0.01	0.02	0.73	0.47	0.03 (− 0.03 to 0.08)	0.03	0.03	0.95	0.34	− 0.03 (− 0.06 to 0.00)	.02	− 0.06	− 2.24	0.03	.00(− .01 to .02)	0.01	0.01	0.44	0.66
RT sad	− 0.00 (− 0.03 to 0.02)	0.01	− 0.01	− 0.23	0.82	0.02 (− 0.05 to 0.07)	0.03	0.02	0.48	0.63	− 0.00 (− 0.03 to 0.03)	0.02	− 0.01	− 0.15	.88	− 0.00 (− 0.02 to 0.02)	0.01	− 0.00	− 0.12	0.91
RT angry	− 0.01 (− 0.04 to 0.01)	0.01	− 0.04	− 1.07	0.29	− 0.03 (− 0.09 to 0.03)	0.03	− 0.03	− 0.94	0.35	− 0.01 (− .05 to 0.02)	0.02	− 0.02	− 0.69	0.49	− 0.00 (− 0.02 to 0.02)	0.01	− 0.00	− 0.07	0.94
RT fear	− 0.00 (− 0.03 to 0.02)	0.01	− 0.00	− 0.12	0.91	− 0.03 (− 0.09 to 0.03)	0.03	− 0.04	− 1.04	0.30	0.01 (− 0.02 to 0.04)	0.02	0.02	0.70	0.48	− 0.02 (− 0.03 to 0.00)	0.01	− 0.05	− 1.63	0.10
RT disgust	0.02 (− 0.01 to 0.04)	0.01	0.04	1.21	0.23	0.04 (− .02 to 0.10)	0.031	0.04	1.17	0.24	− 0.00 (− 0.03 to 0.03)	0.02	− 0.00	− 0.07	0.95	0.02 (.00 to 0.04)	0.01	0.06	2.09	0.04

n = 1956–2059

All effects were adjusted for sex, age, mental health problems at age 11. P-values in bold indicate significance after Bonferroni–Holm correction

*C.I*. confidence interval, *RT* reaction time (standardized), *EP* error proportion (standardized)

### Post-hoc Exploration: The 5% Lowest Scores on Facial Emotion Identification Abilities and the Frequency of Psychotic Experiences

We hypothesized that perhaps only adolescents who scored very poorly on facial emotion identification at preadolescence were more vulnerable for developing psychotic experiences at adolescence. Therefore, to investigate whether a specific subsample, namely preadolescents with the lowest scores (highest 5% of EPs and longest 5% of RTs) on the facial emotion identification task are at an increased risk for psychotic experiences in adolescence, we conducted a post hoc exploration. The group of 5% lowest scorers on the emotion perception task had an average EP (%) of 13.67 (SD: 1.64), 38.46 (SD: 3.42), 24.00 (SD: 4.59), 27.20 (SD: 4.76) and 20.62 (SD: 3.41), for the emotions happy, sad, angry, fear and disgust respectively. The mean RTs (ms) for this group were 1431 (SD: 126), 1990 (SD: 157), 1805 (SD: 139), 1840 (SD: 173), 1719 (SD: 138), for the emotions happy, sad, angry, fear and disgust, respectively. We compared the lowest 5% with the remaining 95% of scores of EPs and RTs on all emotions (happy, sad, angry, fear and disgust) at preadolescence on the frequency of psychotic experiences at adolescence using independent samples t-tests, finding no significant differences between the groups (see the supplementary table for more information).

## Discussion

Reduced social cognition has often been identified as a trait marker for psychosis, as it is compromised in early phases of psychosis [10], as well as in siblings of individuals diagnosed with a psychotic disorder [13]. We examined whether diminished facial emotion identification can be identified as a vulnerability marker for subsequent psychotic experiences in a young adolescent sample. The results did not confirm our hypothesis that facial emotion identification abilities at preadolescence were associated with psychotic experiences at adolescence. When examining a sub-sample of preadolescents scoring the lowest performance on the facial emotion identification task, we still found no vulnerability for psychotic experiences associated with impaired identification of facial affect in adolescence. In absence of an association, mediation by family factors was not explored. As a main effect, increased overprotective parenting at preadolescence was associated with a higher frequency of psychotic experiences as well as delusions in adolescence, after adjustment for preadolescent mental health. There was no indication that parenting stress, family functioning, and rejective and warm parenting were associated with psychotic experiences, indicating these factors may not pose a vulnerability for psychotic experiences.

In the broader adolescent population, when individuals are not recruited for their high risk status or previous episode of psychosis, facial emotion identification does not seem to be predictive of the development of psychotic experiences. Thus, it is possible that the association between facial emotion identification and psychotic experiences is not present in a large and relatively healthy sample. We speculated that perhaps this association would be detectable in a subgroup of adolescents with demonstrably lowered performance in facial emotion identification. However, a post hoc examination based on this subsample also showed no indication of a vulnerability for psychotic experiences over time. Although the reporting of psychotic experiences may increase the risk of developing a mental illness [21, 64–66], the large majority of psychotic experiences are transient and benign during adolescence [67]. Therefore, perhaps an impairment in facial emotion identification is not predictive of psychotic experiences in adolescence, but it may be predictive of clinical psychotic symptoms in young adulthood. This reasoning would be in line with findings of a recent study [68] which reported that developmental cognitive deficits between infancy and adulthood are only found in those who develop a psychotic disorder, with only weak evidence for individuals who have psychotic experiences. The same might hold for the association between facial emotion identification abilities and family functioning, which perhaps becomes evident only at levels of actual impairment.

In the current study, perceived overprotective parenting at preadolescence was predictive of the frequency of psychotic experiences at adolescence, after controlling for early existing mental health problems. It should be noted first that we need to be cautious about the clinical relevance of this finding: the effect of overprotective parenting on the frequency of psychotic experiences was relatively small (denoted by the small, but significant correlation and regression coefficient). Second, we need to be cautious about the interpretation. It is possible that when parents overly protect their child, the child is less able to form its own coping mechanisms towards daily stressors. As a result, the child may be less resilient to negative events in life, rendering them more vulnerable to develop psychotic experiences and/or delusions. Another explanation may be that overprotection by the parent is a natural reaction towards a child that is more vulnerable, and requires extra support and care. The parent may sense that the child is sensitive towards certain experiences, and the overprotective parenting may then be an attempt of preventing negative outcomes. However, given that the association was corrected for preadolescent mental health problems, this explanation could be less likely. Overprotective parenting may be a trans-diagnostic risk factor, as previous studies have also found overprotective parenting to be predictive of substance abuse [69, 70], anxiety [71], and internalizing and externalizing problems [72]. Such a risk factor may actually be genetically mediated, which leaves a third explanation that genetic background is causal in both overprotective parenting and in offspring liability to mental health problems. Future research should aim at furthering our understanding of the mechanisms shaping the association.

We expected that rejective parenting, parenting stress, lower family functioning and a lack of warm parenting would also predict psychotic experiences in adolescence, but we did not find evidence for this in the current study. It is possible that overprotective parenting is specifically relevant for the development of psychotic or internalizing problems, whereas rejective parenting may be more relevant for, for example, aggressive problems [73]. An alternative explanation could be that the negative impact of family factors during preadolescence can be compensated with protective factors in adolescence, such as a strong social network of peers. Indeed, previous findings demonstrate that although negative parenting (specifically dominant and harsh parenting) is predictive of externalizing behaviors in adolescence, the association was attenuated by good quality friendships and peer group affiliation [74]. In contrast, overprotective parenting often renders a child placid, cautious and sensitive [75], making them less attractive to peers, and more often at risk of peer victimization [76]. Future research could examine whether the protective effect of peer relationships on negative parenting in preadolescence is less strong (or perhaps not evident) for overprotected children.

This study has a number of limitations. The Facial Expressions Task (ANT [39] is not suited to assess biases in facial emotion identification. An emotional bias is a qualitative deviation in emotional processing [77], such as for example, the under-attribution of happiness when labelling neutral faces [7]. Given that previous studies have found that emotional biases are present and important in psychosis [78, 79] our study would have been more comprehensive to assess biases in addition to the ability to identify emotions per se. In addition, the inclusion of neutral faces would have yielded more information, as processing of neutral faces (a socially ambiguous stimulus) has reported to be abnormal in individuals with a psychotic disorder [80]. A further limitation of our study is the lack of a control group of adolescents with clinical psychotic symptoms, as this would allow us to test our hypothesis that a facial emotion identification vulnerability may only be associated with clinical psychotic symptoms, rather than psychotic experiences more generally. In addition, having knowledge on the family history of mental health problems could shed more light on the potential presence of a genetic liability for both overprotective parenting and psychotic experiences. Last, in the ideal design, we would have assessed psychotic experience at age 11 (rather than general problem behavior), as well as emotion identification at age 16, which would have allowed us to examine concurrent associations that aid in the interpretation of our null findings across these 5 years.

This study also has a number of strengths. First, we used a longitudinal design to examine whether facial emotion identification and family factors would predict psychotic experiences in adolescence, where most studies utilize cross-sectional designs (or shorter follow-up periods) and examine these associations in older samples or in samples with individuals who already have psychotic experiences or symptoms, thus limiting the examination of cause-consequence associations. Second, our study has a large sample size and a follow-up period of 5 years. To the best of our knowledge, we were the first to examine in a longitudinal way whether preadolescent facial emotion identification abilities and family factors have the potential to predict psychotic experiences in adolescence.

The current study examined whether facial emotion identification and family factors at preadolescence (age 11) were predictive of psychotic experiences 5 years later at adolescence (age 16). Facial emotion identification at preadolescence was not associated with psychotic experiences at adolescence. This may suggest that a facial emotion recognition vulnerability for psychosis cannot be detected in early adolescence. Alternatively, it may only be evident in subgroups of individuals who ultimately develop a psychotic disorder, indicating that psychotic experiences in adolescence are still too mild or have little specificity for the subsequent psychotic disorder. Overprotective parenting at preadolescence predicted the frequency of both psychotic experiences and delusions, after adjusting for preadolescent mental health. Possibly, overprotective parenting at a young age results in a lack of self-reliance, autonomy or coping skills in adolescents, making them especially vulnerable to psychotic experiences as a reaction to life stressors. However, it could be that overprotection by parents is a natural reaction towards a child that is more vulnerable, and requires extra support and care. Likewise, overprotection by parents and their children’s vulnerability for psychotic experiences could have a shared background, for example, a shared genetic liability. Future research is needed to examine the mechanism behind the role of overprotective parenting on psychotic experiences during adolescence.

## Summary

An impairment in facial emotion identification could signify a vulnerability for the development of psychosis. Family functioning may mediate the association between facial emotion identification and psychotic experiences. The current study examines whether facial emotion identification and family factors at preadolescence (age 11) predict psychotic experiences 5 years later during adolescence (age 16). Data was obtained from the epidemiological cohort TRAILS (TRacking Adolescents’ Individual Lives Survey; N = 2059). At preadolescence, a facial emotion identification test and three questionnaires to assess family functioning, perceived parenting styles and parenting stress, were administered. At adolescence, a questionnaire on psychotic experiences was administered. Data were analyzed using multiple linear regression models. Facial emotion identification at preadolescence was not associated with psychotic experiences at adolescence, and the mediational role of family functioning was therefore not further explored. Increased overprotective parenting at preadolescence was associated with a higher frequency of psychotic experiences and delusions at adolescence, while the other family factors (parenting stress, family functioning, and rejective and warm parenting) at preadolescence were not significantly associated with psychotic experiences at adolescence. While clinical symptoms in early and chronic psychosis have been associated with facial emotion identification deficits, this association was not present in the current adolescent cohort. Conversely, perceived overprotective parenting was prospectively associated with psychotic experiences, possibly either due to a vulnerability for psychosis, a natural reaction towards a vulnerable child, or a shared genetic liability in both parents and adolescents. Future research may examine the mechanism behind the role of overprotective parenting on psychotic experiences during adolescence.
